# *In silico *identification of putative promoter motifs of White Spot Syndrome Virus

**DOI:** 10.1186/1471-2105-7-309

**Published:** 2006-06-19

**Authors:** Hendrik Marks, Xin-Ying Ren, Hans Sandbrink, Mariëlle CW van Hulten, Just M Vlak

**Affiliations:** 1 Laboratory of Virology, Wageningen University, Binnenhaven 11, 6709 PD Wageningen, The Netherlands; 2 Plant Research International, Postbus 16, 6700 AA, Wageningen, The Netherlands; 3 NCMLS/Radboud University Nijmegen, Department of Molecular Biology, Geert Grooteplein 26/28, 6525 GA, Nijmegen, The Netherlands; 4 CSIRO Livestock Industries, 306 Carmody Road, St Lucia 4067, Brisbane, Australia

## Abstract

**Background:**

White Spot Syndrome Virus, a member of the virus family *Nimaviridae*, is a large dsDNA virus infecting shrimp and other crustacean species. Although limited information is available on the mode of transcription, previous data suggest that WSSV gene expression occurs in a coordinated and cascaded fashion. To search *in silico *for conserved promoter motifs (i) the abundance of all 4 through 8 nucleotide motifs in the upstream sequences of WSSV genes relative to the complete genome was determined, and (ii) a MEME search was performed in the upstream sequences of either early or late WSSV genes, as assigned by microarray analysis. Both methods were validated by alignments of empirically determined 5' ends of various WSSV mRNAs.

**Results:**

The collective information shows that the upstream region of early WSSV genes, containing a TATA box and an initiator, is similar to *Drosophila *RNA polymerase II core promoter sequences, suggesting utilization of the cellular transcription machinery for generating early transcripts. The alignment of the 5' ends of known well-established late genes, including all major structural protein genes, identified a degenerate motif (ATNAC) which could be involved in WSSV late transcription. For these genes, only one contained a functional TATA box. However, almost half of the WSSV late genes, as previously assigned by microarray analysis, did contain a TATA box in their upstream region.

**Conclusion:**

The data may suggest the presence of two separate classes of late WSSV genes, one exploiting the cellular RNA polymerase II system for mRNA synthesis and the other generating messengers by a new virus-induced transcription mechanism.

## Background

White Spot Syndrome Virus (WSSV), type species of the virus family *Nimaviridae *(genus *whispovirus*), is a pathogen of major economic importance in cultured penaeid shrimp [1,2]. Histopathological studies on WSSV infected shrimp have shown that the virus mainly infects tissues of ectodermal and mesodermal origin, such as the stomach, gills, heart, gut, muscle tissue and hematopoietic tissue [3-5]. Infected cells within these tissues are characterized by the appearance of homogeneous hypertrophied nuclei and chromatin margination [1,5,6]. WSSV particles have been mainly detected in the nuclei of infected cells, indicating that transcription, replication and virion assembly probably occur in the nucleus [5-8]. It is not clear how the virions are released from the nucleus of an infected cell, but this most likely occurs by budding or by rupture of the nuclear envelope and/or the cell membrane.

The circular ds DNA genome of three WSSV isolates, originating from Taiwan (WSSV-TW), China (WSSV-CN) and Thailand (WSSV-TH), have been completely sequenced [9-11]. The genome of WSSV-TH has a size of 292,967 bp encompassing 184 open reading frames (ORFs), which are almost equally distributed on both strands [10]. Thus far, no evidence has been obtained for the occurrence of spliced transcripts. Only a limited amount of the WSSV ORFs could be assigned a function based on homology with known genes in public databases [10]. Concerning genes involved in replication and transcription of WSSV, four putative functional proteins have been annotated: a DNA helicase (ORF9), a DNA polymerase (ORF27), a cAMP-responsive element binding protein (ORF66) and a TATA box binding protein (ORF149). Furthermore, several genes involved in nucleotide metabolism, such as both subunits of a ribonucleotide reductase, a chimeric thymidine-thymidylate kinase, a thymidylate synthase, a dUTPase and an endonuclease have been identified on the genome [10,11]. Although present in various other large dsDNA viruses, no RNA polymerase or other genes involved in transcription, e.g. a poly(A)polymerase or mRNA capping enzymes, have (yet) been identified on the WSSV genome. Around 50 major or minor virion protein genes have been identified on the genome [12-15].

Upon infection, expression of the WSSV genes can be divided in at least an early and a late phase [16], while also an immediate-early phase might be present [17]. The mechanism of the switch between (immediate-) early and late WSSV gene expression, as well as the promoters and regulatory sequences involved, is largely unknown. However, many eukaryotic large ds DNA viruses of >100 kb have a coordinated and cascaded fashion of gene expression [18-21]. Baculoviruses and herpesviruses (both replicating in the nucleus) as well as poxviruses and asfarviruses (both replicating in the cytoplasm) express their early genes before viral replication initiates, while late genes are expressed after the onset of viral DNA replication. Both viruses replicating in the nucleus utilize the host RNA polymerase II for early gene transcription [19,22]. However, for late gene transcription, herpesviruses continue to exploit the cellular RNA polymerase II system, while late transcription of baculoviruses occurs by a novel RNA polymerase that is at least partially encoded by the baculovirus genome [22-24]. The viruses replicating in the cytoplasm encode their own RNA polymerase which synthesizes early as well as late mRNAs. This RNA polymerase is encapsidated within the virus particle to enable the initiation of viral gene expression upon arrival in the cytoplasm [18,25].

Despite the differences in gene expression strategies, the above viruses have in common that specific nucleotide motifs involved in transcription initiation, expression kinetics and expression level have been identified in the upstream regions of individual genes. Well known promoter elements used by many viruses are the TATA box and the initiator sequence, which is located at or near the site of transcription initiation (TIS). We hypothesize that conserved promoter motifs play an important role in transcription regulation of WSSV, and that they can be identified by *in silico *analysis of upstream regions of WSSV genes. As important promoter motifs are overrepresented in the 5' upstream regions of baculoviruses genes [26], we studied the relative abundance of all 4 through 8 nucleotide motifs in the upstream regions of WSSV genes compared to the complete WSSV genomic sequence. This enumeration strategy was validated by testing the eukaryotic large ds DNA viruses mentioned above. To further identify regulatory elements, the nucleotide composition in the upstream regions of WSSV early and late genes, as assigned by microarrays [16], is studied using MEME [27]. MEME is an algorithm which searches for conserved motifs in a selected set of sequences, in this case the upstream regions of WSSV ORFs. Experimental support for the *in silico *results is obtained by alignments of 5' ends of known WSSV early as well as late transcripts. These alignments include TISs mapped by 5'RACE (Rapid Amplification of cDNA Ends) in previous studies, as well as two newly determined TISs of the major structural protein genes ORF112 and ORF160. Polyadenylation of WSSV early and late genes is studied by alignment of poly(A) sites. Using this approach, we were able to find further support for the presence of coregulated clusters of WSSV genes, as well as to predict putative WSSV promoter elements involved in gene expression of these clusters.

## Results

### Promoter analysis using the enumeration method

In a search for putative WSSV regulatory promoter elements, we compared the abundance of all 4, 5, 6, 7 or 8 nucleotide motifs in the 100 and 200 nt upstream sequences of all WSSV genes relative to their presence in the complete WSSV genomic sequence. This method will be referred to as the enumeration method in the remaining part of the article. For validation, this enumeration method was applied on the genome sequences of the type species of more extensively studied large ds DNA viruses mentioned in the introduction: *Ac*MNPV (*Autographa californica *Multinucleopolyhedrovirus; Baculovirus), Human Herpes Virus 1 (HHV1; Herpesvirus), Vaccinia virus (Poxvirus) and African Swine Fever Virus (ASFV; Asfarvirus).

#### *Ac*MNPV, HHV1, Vaccinia virus and ASFV

Only the analysis of the 4-mers of these viruses is shown, as these will always be included in larger motifs (Table 1). Most *Ac*MNPV early genes contain a functional consensus TATA box upstream of the TIS [19]. *Ac*MNPV initiator motifs are composed of the conserved nucleotide sequence CAGT and (a/g/t)TAAG, for early and late genes, respectively [18,23,28]. Ayres *et al*. [26] showed that the sequence TAAG occurs less frequently in the whole *Ac*MNPV genome than expected based of the *Ac*MNPV nucleotide composition. The results of the 4-mer motif frequency in the 100 nt upstream of all *Ac*MNPV ORFs analyzed with the enumeration method indeed shows that the TAAG motif frequency is 29% of the expected occurrence in the whole genome (Table 1). However, the analysis also shows that this motif has the highest relative enrichment in the upstream regions of the *Ac*MNPV ORFs of all possible 4-mer motifs (4.0 times). Also the baculovirus early promoter motif CAGT is relatively more frequently present in upstream regions (1.4 times), although not as prominent as the TAAG motif. Parts of the TATA box as well as sequences of the well known baculovirus early transcription activating motifs GATA and CACNG [19] occur relatively often in the upstream regions of the ORFs (Table 1). Compared to 100 nt, the enrichment of the functional motifs in 200 nt upstream of the *Ac*MNPV ORFs is less pronounced (Table 1) supporting the experimental observation that in baculoviruses important promoter elements are often located within 100 nt upstream of the translational start codon [26]. Analysis of 5-mer motifs of *Ac*MNPV revealed that (a/g/t)TAAG was enriched in the upstream regions of the ORFs, but not CTAAG. Analysis of 6-mer motifs showed a relative enrichment of 3.0 times of the consensus TATA box sequence TATAAA in the 200 nt upstream regions.

For HHV1, the 4-mer nucleotide motifs of known promoter elements were identified by the enumeration method during analysis of the 200 nt upstream sequences, but not when analyzing 100 nt upstream sequences. This supports the view that, in contrast to baculoviruses, most regulatory elements are located more than 100 nt upstream of the HHV1 translational start codons [21]. Parts of the consensus TATA box, involved in HHV1 early and late transcription [21,29], occur relatively frequently in the 200 nt upstream of the HHV1 ORFs (Table 1). Also the sequence CATT, part of the CCATT boxes which are typically located upstream of the consensus TATA box of HHV1 early genes [21], shows a high relative enrichment of 2.1 (Table 1).

For both cytoplasmatic viruses Vaccinia virus and ASFV the analysis shows that the late initiator sequences, TAAAT and TATA respectively [18,20,30], are highly enriched in the 100 nt as well as the 200 nt upstream sequences, although not as prominent as the late TIS of baculoviruses (Table 1). Also parts of the sequence TAAA(a/t), essential for Vaccinia virus intermediate gene expression, are enriched (Table 1). Furthermore, the analysis shows a considerable enrichment of motifs only consisting of A and T residues. Long stretches of these nucleotides upstream of the transcribed region are typical for Vaccinia virus and ASFV early promoters, as well as for ASFV late promoters [18,20,30].

#### WSSV

The same enumeration method was used to analyze the upstream sequences of WSSV ORFs. The analysis of the 4- and 5-mer motifs is shown in Table 1. Sequences of the consensus TATA box appear relatively frequently compared to their presence in the complete WSSV genome (Table 1). The enrichment of these TATA box sequences is similar to what is observed for *Ac*MNPV and HHV1 (Table 1), indicating a functional role for the TATA box in WSSV transcription regulation. Besides the TATA box sequences, the sequence AACC has the highest enrichment in the 100 nt upstream sequences of WSSV ORFs, although not as pronounced as the occurrences of the *Ac*MNPV TAAG motif (Table 1). Previous experiments showed that the TISs of the late WSSV envelope protein genes *vp28 *and *vp19 *start within this exact AACC sequence [31] indicating this could be a putative promoter element for late transcription. Furthermore, some motifs consisting of G and C residues, such as the 4-mers CCGG and CCCC and the 5-mers CCGGG and CCCGG (Table 1), and G/C-rich sequences have a relatively high frequency in WSSV upstream regions. Compared to the 100 nt upstream of the ORFs, the results for the analysis of 200 nt are only slightly different and mostly less pronounced (Table 1). From the remaining analysis using 6, 7 or 8 nt motifs in the 100 or 200 nt upstream regions (data not shown), it is noteworthy that the enumeration method shows a relative enrichment of the 6-mer consensus TATA box sequence TATAAA of 4.7 times in the 100 nt upstream of the ORFs.

Previously, we showed that the WSSV genes clustered in an early and a late class based on expression profile in shrimp tissue [16]. Further analysis within the 100 nt upstream regions of either the WSSV early or late genes using the enumeration method showed that the sequence AACC has the highest relative enrichment of all possible 4-mer motifs for the late genes (2.4 times), while sequences of the TATA box were highly enriched in upstream regions of both gene classes (the sequence TATA showed a relative enrichment of 2.3 times in the 100 nt upstream regions of both gene classes; other data not shown).

### MEME

The 100 or 200 nt sequences upstream of all WSSV genes were also studied by MEME (Table 2). As multiple classes of coregulated viral genes will be present within these sequences, the MEME settings for this analysis were to identify conserved motifs regardless whether it occurred in the upstream regions of all genes. MEME identified the TATA box as consensus nucleotide motif in these WSSV upstream sequences (Table 2). Furthermore this analysis showed that multiple upstream sequences contain stretches of T residues (Table 2). Analysis on the location and composition of these sequences revealed that these are mostly part of the polyadenylation signals [32] of the upstream ORFs, and therefore probably not functional as promoter element of WSSV. The outcome of the 100 and 200 nt upstream sequences are very similar, in line with the results of the enumeration method.

For individual analysis of the WSSV early or late kinetic cluster [16] the frequency of a specific motif per individual sequence was set at one, as most WSSV genes belonging to one cluster were considered to be coregulated. Analysis of the 100 and 200 nt upstream regions of either the early or the late class genes identified the consensus TATA box as putative promoter element (Table 2). Previously, we already showed that 37 of the 64 early genes (58%) and 28 of the 58 genes that clustered late (48%) contain a consensus TATA box [16]. Specific for the early class, MEME identified the consensus sequences CAACATCA and AGAAT, while for the late class it identified the consensus sequence AACC as well as an A-rich region (Table 2). On the other hand, as the early or late kinetic cluster [16] could also consist of subsets of coregulated WSSV genes, an additional MEME analysis was performed in which a motif only had to occur in at least half of the upstream sequences of either the early or the late genes. The outcome was very similar to the results presented in Table 2. Interestingly, the TATA box and the AACC motif were identified by the enumeration method as being highly enriched in upstream regions of WSSV ORFs.

### Alignments TISs of WSSV genes

To validate both *in silico *methods described above, we compared the outcome with alignments of all known 5' ends of the WSSV early and late class genes. To facilitate comparisons with other viruses, in these alignments the function of the protein encoded by the gene is used to determine its class, either early or late. Early genes often encode enzymes which have functions involved in processes such as nucleotide metabolism, DNA replication, protein modification, viral transcription initiation and host response modulation. Structural virion protein genes often comprise a large part of viral late genes. For nearly all WSSV genes analyzed, this classification matched the results obtained by the microarray study [16].

#### Early genes

**Figure 1:**
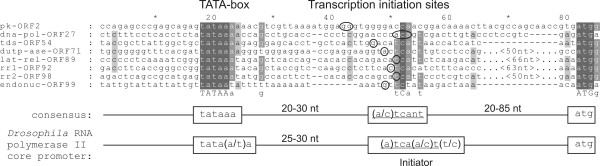
**Alignment of 5' flanking sequences of WSSV early genes**. The genes are named after WSSV-TH ORF numbers and the function of their protein product. The transcription initiation site of each gene is encircled. Sequences are aligned by their consensus TATA box, as well as by maximizing the identities around the transcriptional start site. Below, the consensus sequence of the alignment and the *Drosophila *RNA polymerase II core promoter are shown. Similar sequences of the consensus TIS motif and the initiator of the *Drosophila *RNA pol II core promoter are underlined. Abbreviations used and references: *pk*: protein kinase [52]; *DNA-pol*: DNA polymerase [47]; *tds*: Thymidylate Synthase [48]; *dutp-ase*: dUTPase [42]; *lat-rel*: latency related gene [33]; *rr1 *and *rr2*: the large and small subunit of ribonucleotide reductase, respectively [53]; *endonuc*: endonuclease [54].

The WSSV genome encodes around 10 genes which, based on their (putative) function, are considered to be early [10]. For several of these genes the 5' end of their transcripts has been mapped. RT-PCRs and/or Northern Blots of viral time courses confirmed that these genes were expressed in an early stage during infection (for references see Fig. 1). Furthermore ORF89, which is thought to be involved in latency, was empirically shown to be (immediate) early [33,34]. Fig. 1 shows an alignment of the experimentally determined transcription initiation sites (TISs) of WSSV early genes. The genes typically contain a consensus TATA box (sequence: TATA(a/t)A) [35]. The TIS is located 20 to 30 nucleotides downstream of the consensus TATA box, which is considered to be a functional distance [35-37]. This is between 20 to 85 nucleotides upstream of the translational start codon of the early gene products (Fig. 1). When the sequences are aligned by maximizing the identities around the transcriptional start site (Fig. 1), a clear consensus transcription initiation motif ((a/c)TCANT) overlapped with the transcriptional start sites. This resembles the RNA polymerase II core promoter motif identified in *Drosophila*, which often consists of a consensus TATA box and/or an initiator with the sequence (A)TCA(+1)(g/t)T(t/c) [35-38]. Similar to WSSV, the initiator of *Drosophila *is typically located 25–30 nt downstream of a TATA box [35-37]. Interestingly, the motif CTCAC, which is part of the identified WSSV consensus sequence (a/c)TCANT and which is the exact sequence of the TISs of the *dutpase *and *rr1 *(Fig. 1), was also shown to be enriched in upstream regions of WSSV ORFs (Table 1).

#### Late genes

**Figure 2:**
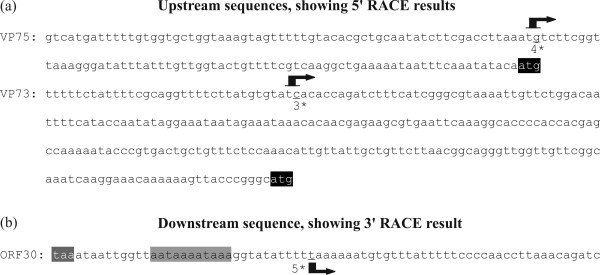
**Sequences upstream of two major structural protein genes *vp75*(ORF160) en *vp73 *(ORF112) of WSSV showing the transcription initiation sites of both genes**. The TISs are indicated by arrows above the sequences. The 5' termini of the different clones sequenced for each gene are underlined. The number beneath the underlining shows the number of similar clones. The start codons of both genes are shaded black (**A**). Sequence downstream of ORF30 showing the polyadenylation site, indicated by an arrow below the sequence. The 3' terminus of the different clones sequenced is underlined. The number before the arrow represents the number of similar clones sequenced. The stop codon is shaded dark grey and two overlapping poly(A) signals (AATAAA) light grey (**B**).

The protein pattern of WSSV particles on an SDS-PAGE gel shows around 8 major WSSV structural virion proteins [12-15]. For 6 of these proteins (VP664, VP28, VP26, VP24, VP19 and VP15) the 5' end of the corresponding mRNA has been mapped [31,39]. RT-PCRs and/or Northern Blots of viral time courses confirmed that these genes were expressed in a late stage during infection [31,39]. We completed this analysis by mapping the TISs of the two other major structural protein genes, *vp75 *(ORF160) and *vp73 *(ORF112). Both *vp75 *and *vp73 *lack a consensus TATA box (Fig. 2a). Using 5'RACE, the TIS of *vp75 *was identified within the nucleotide sequence TG, 72 nt upstream of the translational start codon. For *vp73*, the TIS was located at nucleotide residues TC, 220 upstream of the translational start codon (Fig. 2a).

**Figure 3:**
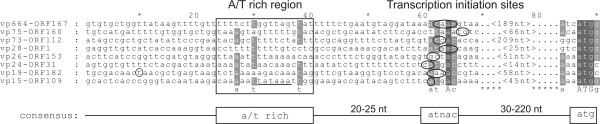
**Alignment of 5' flanking sequences of WSSV late genes**. Name of structural protein genes as well as WSSV-TH ORF numbers are indicated. The transcription initiation site of each gene is encircled. For *vp19 *a minor transcription initiation site is also encircled. The TATA box for *vp15 *is underlined. The A/T rich region is boxed. Sequences are aligned by maximizing the identities around the transcriptional start site. References: *vp28*, *vp26*, *vp24*, *vp19 *and *vp15 *[31]; *vp664 *[39]; *vp75 *and *vp73 *(this study).

When the upstream sequences of all major structural protein genes are aligned by maximizing the identities around the transcriptional start sites (Fig. 3), the TISs are present within or very near the nucleotide sequence ATNAC. The transcripts start 20–25 nucleotides downstream of an A/T rich region, which has an average A/T content of 79% compared to 61% of the 200 nt upstream regions of the 8 genes. *Vp15 *and *vp19 *contain a consensus TATA box, of which only the TATA box of *vp15 *is at a functional distance of the TIS (Fig. 3) [31]. The length of the TIS to the translational start codon is different for the various genes, ranging from 30 to 220 nt (Fig. 3). Interestingly, most of these features were predicted by our *in silico *analysis. The first three nucleotides of the AACC motif identified in the *in silico *analysis (Tables 1 and 2) are part of the consensus sequence ATNAC, and both contain the AC dinucleotide which is present for almost all genes in Fig. 3. Also the sequences ATAA and TAAC, parts of the ATNAC sequence, were identified as putative promoter elements (Table 1). Of all WSSV late genes, as assigned by microarray analysis [16], 40% (23 of the 58, both structural and non-structural protein genes) contains the sequence ATNAC in their 100 nt upstream region. The A-rich (and T-rich) sequences identified by MEME are in line with the observation that late genes often contain long stretches of A/T residues upstream of their TIS (Fig. 3).

In addition to the 8 major structural proteins, the protein profile of WSSV particles shows a range of about 40 minor virion proteins [12,13]. Most of these have not been studied in detail. However, the corresponding messengers are supposed to be late, although 13 of them clustered in the early class during microarray analysis [16]. Remarkably, 45% of the minor virion protein genes (18 of the 40) contain a consensus TATA box within 300 nt of the translational start codon. This is in line with the MEME analysis, which also suggested that the TATA box might be involved in late transcription.

### Polyadenylation

For various WSSV genes, the site of polyadenylation has been mapped using 3'RACE. We extended this analysis by mapping the polyadenylation site of ORF30, the collagen-like ORF of WSSV [40]. Polyadenylation of ORF30 starts 32 nt after the translational stop codon, 16 nt after the first poly(A)-signal (sequence AATAAA; Fig. 2b) [41].

**Figure 4:**
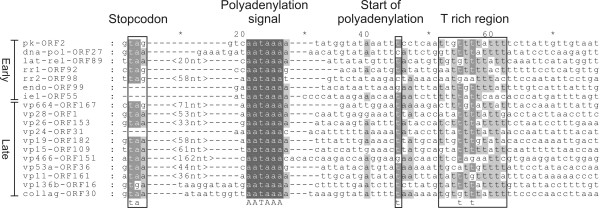
**Alignment of 3' flanking sequences of WSSV genes**. The stop codon, polyadenlylation signal, start of polyadenylation and the T rich region are indicated. Sequences are aligned by stop codon and by polyadenylation signal. Abbreviations used and references: *ie1*: immediate-early 1 [17]; *vp466 *[12]; *vp53a*, *vp11*, *vp136b *[13]; *collag*: collagen-like ORF (this study). For abbreviations and references of other genes see Figs. 1 & 3.

Fig. 4 shows an alignment of all known polyadenylation sites of WSSV. Polyadenylation typically starts within 11–19 nt after a consensus polyadenylation site. Typically, a T rich region (stretch of about twelve T residues) was identified 8 nt downstream of the poly(A)-site (Fig. 4). There seems to be no difference between the polyadenylation sites of early and late genes (Fig. 4). A total of 9 WSSV genes were found to be non-polyadenylated [13,42]. Except for *vp12a *(WSSV-TH ORF34), all these genes lack a consensus poly(A)-signal within -50 to 300 nt of their translational stop codon. Two (*vp31 *and *vp13b *encoded by WSSV-TH ORF163 and ORF155, respectively) do however contain the sequence ATTAAA within this region, which in vertebrates is often sufficient for polyadenylation [43], but apparently not in invertebrates or arthropods.

## Discussion

In this paper, we used a new enumeration strategy based on a model proposed by Brazma *et al*. [44] to identify putative WSSV promoter elements. A set of computer scripts was designed, which calculated the difference in nucleotide motif frequencies in the upstream sequences of all genes compared to the complete WSSV genomic sequence. The rationale behind this analysis is that promoter motifs are often thought to be transcription factor binding sites, which are functional upstream of genes. The results obtained with the well studied large ds DNA viruses *Ac*MNPV, HHV1, Vaccinia virus and ASFV (Table 1) show that our method is robust in identifying important promoter elements of completely sequenced viral genomes without *a priori *knowledge, as these are often enriched in upstream sequences of viral ORFs. Therefore, this new enumeration method can be useful in the analysis of newly sequenced genomes of large ds DNA viruses. For further analysis of the upstream regions of WSSV genes of the early and late cluster, as assigned by microarray analysis [16], MEME was used. Genes of either cluster might be coregulated by similar mechanisms, utilizing conserved nucleotide motifs. As MEME can identify motifs which have to occur in each individual sequence of a set of submitted sequences, or in a selected number of submitted sequences, it is highly complementary to the enumeration method. Another advantage of MEME is that it can identify degenerate motifs.

The enumeration method identified various nucleotide motifs (Table 1) that were also identified by MEME (Table 2) and by the alignments of experimentally determined 5' ends of WSSV mRNAs (Figs. 1 and 3). These include the consensus TATA box, as well as the nucleotide motif AACC. However, also other nucleotide motifs that were not validated with the other methods, e.g. some motifs rich in C or G residues, were (highly) enriched in WSSV upstream regions and might be involved in WSSV transcription. In accordance with the alignments shown in Figs. 1 and 3, where most putative promoter elements are located within 100 nt upstream of the ORFs, the nucleotide motifs identified with the enumeration method are most pronounced in the 100 nt upstream of the ORFs (Table 1) compared to 200 nt. This suggests that, similar to *Ac*MNPV, most WSSV promoter elements are located within 100 nt upstream of the translational start codons, which is a reflection of the tight package of genes along the WSSV genome. It would be of interest to test the functionality of the sequences (a/c)TCANT and ATNAC, which were identified as the consensus TISs of the WSSV early and late class genes, respectively (Fig. 3) and other identified motifs (Table 1) in a reporter gene (e.g. luciferase) assay. For testing late promoters in this setup, a co-infection with WSSV should be considered to supply additional viral transcription factors required for late gene expression. In the absence of a suitable WSSV cell system, these reporter gene assays have been performed in the artificial Sf9 insect cell line [17,33,45] with all its limitations to the interpretation of the results. However, with the recent developments concerning differentiation and growth of crayfish hematopoietic stem cells *in vitro *[46], these experiments might be performed in crayfish cell cultures providing a more convenient and homologous system.

The identification of (putative) promoter elements provides further insight in the transcription mechanisms used by WSSV. The presence of a consensus TATA box for most early genes as well as a conserved transcription initiation motif similar to the *Drosophila *initiator suggest that WSSV uses the host RNA polymerase II transcription machinery for generating early transcripts, as also proposed by Chen *et al*. [47] and Liu *et al*. [42]. Previous analysis of WSSV late genes could not reveal any readily apparent dominant nucleotide element used for WSSV late gene expression [31]. Using the newly available microarray clustering [16], we could now show that around half of the WSSV putative late genes contain a consensus TATA box. This suggests that WSSV might exploit the cellular RNA polymerase II system not only for early but also for (part of) its late mRNA synthesis, similar to some other ds DNA viruses like herpesviruses [22]. Only one of the 8 major structural virion protein genes, which are expressed in the late phase of viral infection and most likely are co-regulated to secure correct assembly of the virion, contains a consensus TATA box. Alignment of the 5' ends of the 8 major structural protein genes identified a novel consensus transcription initiation site, ATNAC, downstream of an A/T rich region. The *in silico *analysis further supports the observation that both components might be late promoter elements. This suggests a second pathway for WSSV late gene expression, similar to the late gene expression strategy identified for baculoviruses [23,24]. However, different from baculoviruses, viral genes required for this pathway, such as a RNA polymerase or late transcription factors, have not been identified on the WSSV genome [10,11]. These genes could however be too much diverged from known homologues to be found based on amino acid homology.

The alignments of the 3' ends of WSSV mRNAs suggest that there is no difference in polyadenylation between early and late mRNAs. The WSSV polyadenylation characteristics of both classes resemble regular polyadenylation in eukaryotic mRNAs, which is typically located 10 to 25 nt downstream of the sequence AATAAA [41,43]. Also oligo-T stretches are often present about 30 nt downstream of the poly(A)-signal of eukaryotic genes [32]. These data indicate that WSSV uses the regular cellular enzymes for polyadenylation of mRNAs. However, other undefined signal pathways of polyadenylation might also be used, as two WSSV genes (dUTPase and TdS) were found to be polyadenylated without a poly(A)-signal present [42,48].

## Conclusion

Using a combined approach of *in silico *analysis and experimentally determined data on WSSV transcriptomics, further support was found for the presence of different coregulated classes of WSSV genes. Comparisons with other large ds DNA viruses provided insight in the transcription mechanism of these classes and putative promoter motifs involved. In order to determine the functionality of these motifs empirically cell culture systems for shrimp will have to be further developed.

## Methods

### Virus infection

The virus isolate used in this study, known as WSSV-TH (acc.no. AF369029), originates from infected *Penaeus monodon *shrimp obtained in Thailand in 1996 and was treated as described before [15]. Crayfish *Orconectes limosus *was injected intramuscularly with purified WSSV using a 26-gauge needle to initiate infection. Three days post infection (d.p.i.), the crayfish were frozen in liquid nitrogen and stored at -80°C until further use.

### 5' and 3' Rapid Amplification of cDNA ends (5'/3' RACE)

Both 5' and 3' RACE were carried out using a commercial 5'/3' RACE kit (Roche) following the manufacturer's instructions. Total RNA was isolated from the frozen gill tissue of three infected crayfish *O. limosus *(harvested 3 d.p.i.) as described before [31]. In case of the 3' RACE of ORF30, first strand cDNA was synthesized using the oligo(dT) anchor primer. The resulting cDNA was amplified using one specific forward primer (ORF30-RACE-F1: CAGACCCGATTACAGTAGCAG; WSSV-TH location: 48983-49003) and the anchor primer. For the 5' RACE of ORF112 and ORF160, the RACE-R1 primers mentioned below were used for synthesis of the cDNA. This cDNA was purified using the High Pure PCR Product Purification Kit (Roche) and a homopolymeric 3' d(A)-tail was added to the cDNA in a mixture with a total volume of 20 μl, using terminal transferase and dATPs included in the kit. This mixture (5 μl) was used in a PCR, performed with an oligo(dT) anchor primer and a nested RACE-R2 primer (see below). The final products of the 5' and 3' RACE were cloned into the pGEM-T easy vector (Promega) and sequenced. For each 5' and 3' RACE at least 3 clones were sequenced. Primers used for 5' RACE: ORF112-RACE-R1: CGCATATTGTTGTTTGTCGTAG (WSSV-TH location 168230-168209); ORF112-RACE-R2: GACGCGTATCTCAAGTATTCC (WSSV-TH location 168184-168164); ORF160-RACE-R1: CTTGTTGGATTCGGAGCAGTG (WSSV-TH location 240137-240117); ORF160-RACE-R2: GACGGATAATATGGGTGACAAG (WSSV-TH location 240111-240090).

### DNA sequencing and computer analysis

Plasmid clones carrying RACE products were sequenced at the company BaseClear (the Netherlands), using universal M13 forward and reverse primers. Sequence data were analyzed using the software package DNASTAR4.2. All sequences data were edited and aligned in GeneDoc, version 2.6.000 [49].

### Promoter analysis using the enumeration method

A set of computer scripts, made in the computer programming language Perl (see [50] for more information), was designed to analyze all 4, 5, 6, 7 or 8 nucleotide motif frequencies in the 100 and 200 nt upstream sequences of (putative) ORFs to compare these with a complete viral genome. In case of WSSV, the genome of WSSV-TH as annotated by van Hulten *et al*. [10] was used for the analysis. For WSSV and *Autographa californica *Multinucleopolyhedrovirus (*Ac*MNPV), the genes and upstream regions of genes that are partly or completely located in the homologous regions (*hr*s, genomic regions consisting of large tandem repeats; for WSSV this concerns 24 genes, for *Ac*MNPV 3 genes) and the *hr*s itself were excluded from the analysis to avoid the possibility of finding false motifs due to the high homology of the *hr *sequences. As nucleotide motifs with a regulatory function are expected to be present in multiple upstream regions, only motifs that were present in upstream sequences of at least 5 ORFs were analyzed after running the scripts. Scripts were used for the following procedures: (1) extraction of the upstream region before the selected ORFs, both on the + and - strand; (2) calculation of the X-mer nucleotide motif frequencies in the upstream regions; (3) calculation of the X-mer nucleotide motif occurrences in both strands of the viral genome (without *hr*s); (4) calculation of the relative ratio of the same X-mers between the upstream regions and the viral genome; (5) ranking of the relative ratios for each X-mer from high to low; (6) exclusion of motifs present in less than 5 upstream regions. Accession numbers of genomes analyzed were *Ac*MNPV [Genbank: L22858; NC_001623]; Human Herpes Virus 1: HHV1 [Genbank: X14112; NC_001806]; Vaccinia virus [Genbank: M35027]; African Swine Fever Virus: ASFV [Genbank: U18466; NC_001659]) and WSSV [Genbank: AF369029]. Statistical analysis was performed by assuming a normal distribution of the enrichment of the nucleotide motifs for each of the viruses analyzed. P ≤ 0.05 in case the enrichment of a certain motif exceeds two times the standard deviation from the average enrichment.

### MEME

The computer program Motif Elucidation using Maximum Expectation maximization (MEME; available at [51]) was used to search for specific sequence motifs in 100 or 200 nt of the WSSV noncoding sequences upstream of the translational initiation codon. MEME analysis was performed using the sequence of WSSV-TH annotated by van Hulten *et al*. [10]. A search was performed for the 3 best motifs with a length of 4–8 nt. In case of analyzing the upstream sequences of the WSSV ORFs at large, the occurrence of a specific motif per individual sequence was set at zero to one, but the motif had to occur in at least 60 upstream regions. In case of analyzing upstream sequences of WSSV genes belonging to the early or late kinetic clusters, the frequency of a specific motif per individual sequence was set at one. The 5' noncoding regions were categorized according to class of expression [16]: Early (ORF2, ORF8, ORF9, ORF11, ORF12, ORF15, ORF16, ORF23, ORF24, ORF25, ORF29, ORF37, ORF49, ORF53, ORF55, ORF56, ORF58, ORF60, ORF61, ORF66, ORF67, ORF69, ORF70, ORF74, ORF81, ORF85, ORF89, ORF91, ORF92, ORF93, ORF98, ORF99, ORF101, ORF103, ORF107, ORF111, ORF112, ORF115, ORF116, ORF117, ORF125, ORF126, ORF127, ORF131, ORF132, ORF142, ORF145, ORF146, ORF147, ORF152, ORF156, ORF159, ORF160, ORF161, ORF164, ORF165, ORF169, ORF170, ORF171, ORF172, ORF173, ORF177, ORF178, ORF179) and Late (ORF1, ORF3, ORF4, ORF7, ORF10, ORF14, ORF27, ORF28, ORF30, ORF31, ORF32, ORF33, ORF34, ORF35, ORF36, ORF38, ORF39, ORF41, ORF43, ORF44, ORF54, ORF57, ORF65, ORF72, ORF73, ORF75, ORF76, ORF77, ORF79, ORF80, ORF84, ORF90, ORF94, ORF95, ORF100, ORF109, ORF113, ORF114, ORF118, ORF119, ORF120, ORF121, ORF128, ORF129, ORF130, ORF134, ORF135, ORF136, ORF143, ORF148, ORF151, ORF153, ORF157, ORF167, ORF168, ORF182, ORF183, ORF184).

## Authors' contributions

HM participated in the design of the computer scripts for the enumeration method and performed most of the analyses in which the scripts were used, performed the MEME analyses, the virus infections, the 5' and 3' RACE experiments including sequencing, and wrote/revised the manuscript. XYR and HS designed and programmed the computer scripts mentioned above. MCWvH and JMV conceived of the study, participated in its design and coordination and helped to draft the manuscript. All authors read and approved the final manuscript.

## Acknowledgements

This work was supported by Intervet International BV, Boxmeer, The Netherlands. We thank Professor Dr Rob Goldbach for continuous interest and advice.

## Figures and Tables

**Table 1:** **Frequency of 4- or 5-nucleotide motifs in the 5' upstream regions of the ORFs as compared to the complete genome for the viruses *Ac*MNPV, HHV1, Vaccinia virus, ASFV and WSSV.** Only the 15 motifs with the highest relative enrichment are shown for each virus. For *Ac*MNPV, HHV1 and WSSV, sequences that are part of the consensus TATA box (TATA(a/t)A) are underlined, while for Vaccinia virus and ASFV sequences only consisting of A and T residues are italics. * Means P ≤ 0.05

**4/5-mer (motif)**	**Occurrence in genome**^**a **^**(% of expected occurrence**^**b**^**)**		**Occurrence in upstream regions (% of expected occurrence**^**b**^**)**		**Relative enrichment in upstream regions**	**4/5-mer (motif)**	**Occurrence in genome**^**a **^**(% of expected occurrence**^**b**^**)**		**Occurrence in upstream regions (% of expected occurrence**^**b**^**)**		**Relative enrichment in upstream regions**
**100 nt upstream**						**200 nt upstream**					

***Ac*MNPV**											

taag^c^	393	(29)	90	(114)	4.0*	taag^c^	393	(29)	137	(87)	3.0*
tata	1314	(66)	172	(149)	2.3*	tata	1314	(66)	255	(111)	1.7*
ataa	1973	(101)	222	(198)	1.9*	ataa	1973	(101)	363	(162)	1.6*
atat	1616	(81)	170	(147)	1.8*	atat	1616	(81)	268	(116)	1.4*
agta	671	(49)	70	(89)	1.8*	agta	671	(49)	109	(69)	1.4*
aagg	473	(51)	41	(76)	1.5	gata^d^	867	(63)	137	(87)	1.4
gata^d^	867	(63)	74	(94)	1.5	aata	2230	(115)	346	(154)	1.3
cact^e^	612	(64)	52	(95)	1.5	cagt^f^	698	(73)	106	(96)	1.3
aata	2230	(115)	186	(166)	1.4	ctta	393	(28)	59	(37)	1.3
atta	1957	(98)	163	(141)	1.4	tcac^e^	669	(70)	99	(90)	1.3
gtat	949	(68)	79	(98)	1.4	gcta	541	(57)	77	(70)	1.2
cagt^f^	698	(73)	58	(105)	1.4	cccc	190	(42)	27	(52)	1.2
taaa	2716	(140)	222	(198)	1.4	tacc	444	(47)	63	(57)	1.2
aggg	233	(35)	19	(50)	1.4	tagt	737	(53)	104	(64)	1.2
tagt	737	(53)	58	(72)	1.4	cact^e^	612	(64)	86	(78)	1.2

**HHV1**											

ttag	221	(53)	17	(168)	3.2*	ataa	372	(192)	42	(445)	2.3*
ctag	182	(20)	13	(60)	2.9*	tata	306	(159)	32	(342)	2.2*
ctct	658	(76)	38	(180)	2.4*	ctag	182	(20)	19	(44)	2.1*
tagc	365	(41)	21	(97)	2.4*	taaa	454	(234)	47	(498)	2.1*
tcta	199	(49)	11	(111)	2.3*	catt^g^	339	(83)	35	(176)	2.1*
tttt	807	(426)	44	(955)	2.2*	taag	254	(60)	26	(127)	2.1*
tagg	342	(38)	17	(77)	2.0*	ttaa	356	(185)	35	(374)	2.0*
cata	369	(90)	18	(180)	2.0*	ctct	658	(76)	64	(152)	2.0*
ctta	254	(62)	12	(121)	1.9*	ttag	221	(53)	21	(104)	2.0*
ccta	342	(39)	16	(75)	1.9	cata	369	(90)	34	(170)	1.9*
tctc	892	(103)	41	(194)	1.9	ctta	254	(62)	23	(116)	1.9*
ctgt	936	(106)	40	(186)	1.8	tttt	807	(426)	72	(782)	1.8*
ctac	492	(56)	21	(99)	1.8	cctt	795	(92)	68	(161)	1.8
ttcc	990	(114)	42	(199)	1.7	aaat	324	(167)	27	(286)	1.7
cact	433	(50)	18	(85)	1.7	ctat	242	(59)	20	(101)	1.7

**Vaccinia virus**											

*taaa*^h^	4421	(94)	472	(140)	1.5*	gcac	391	(66)	77	(91)	1.4*
*aaaa*^h^	5439	(115)	564	(167)	1.5*	*ataa*	4528	(96)	822	(122)	1.3*
*ataa*	4528	(96)	446	(133)	1.4*	*taaa*^h^	4421	(94)	796	(118)	1.3*
*aaat*^h^	4454	(94)	432	(128)	1.4*	tact	2010	(85)	353	(105)	1.2*
cgcg	318	(107)	30	(141)	1.3*	acta	2179	(92)	373	(111)	1.2*
gggg	171	(57)	16	(75)	1.3*	*aaat*^h^	4454	(94)	755	(112)	1.2
acac	1112	(94)	103	(122)	1.3*	*aaaa*^h^	5439	(115)	911	(135)	1.2
*aata*	5203	(110)	479	(142)	1.3*	tgca	832	(70)	138	(82)	1.2
*ttaa*	3720	(79)	337	(100)	1.3	*aata*	5203	(110)	857	(127)	1.2
tact	2010	(85)	179	(106)	1.3	ctac	1264	(107)	208	(123)	1.2
acta	2179	(92)	190	(113)	1.2	gccg	384	(129)	63	(148)	1.2
*tata*	4748	(101)	411	(122)	1.2	gcga	500	(84)	82	(97)	1.2
tgaa	1917	(81)	165	(98)	1.2	cacg	586	(98)	96	(113)	1.2
ctaa	1830	(77)	157	(93)	1.2	accc	403	(68)	66	(78)	1.2
gtaa	1921	(81)	164	(97)	1.2	cata	2186	(92)	358	(106)	1.2

**ASFV**											

*tata*^i^	2686	(91)	261	(199)	2.2*	*tata*^i^	2686	(91)	405	(154)	1.7*
*ataa*	3146	(107)	278	(214)	2.0*	*ataa*	3146	(107)	432	(166)	1.5*
*aatt*	2634	(89)	231	(176)	2.0*	*aatt*	2634	(89)	357	(136)	1.5*
*taat*	2697	(91)	227	(173)	1.9*	*taaa*	3895	(133)	516	(198)	1.5*
*taaa*	3895	(133)	325	(250)	1.9*	*ttaa*	3376	(114)	447	(170)	1.5*
*aata*	3419	(117)	278	(214)	1.8*	*aata*	3419	(117)	445	(171)	1.5*
*ttaa*	3376	(114)	274	(209)	1.8*	*atat*	3022	(102)	392	(149)	1.5*
*atat*	3022	(102)	238	(182)	1.8*	*taat*	2697	(91)	349	(133)	1.5*
*attt*	3890	(131)	306	(231)	1.8*	*ttat*	3146	(106)	406	(154)	1.5*
*ttat*	3146	(106)	247	(187)	1.8*	*attt*	3890	(131)	495	(187)	1.4*
*ttta*	3895	(131)	296	(224)	1.7*	ctaa	1172	(63)	147	(88)	1.4*
*atta*	2697	(91)	203	(155)	1.7*	*atta*	2697	(91)	337	(129)	1.4*
*tatt*	3419	(115)	249	(188)	1.6*	*tatt*	3419	(115)	426	(161)	1.4*
*aaaa*	6731	(232)	480	(372)	1.6*	*ttta*	3895	(131)	482	(182)	1.4*
*aaat*	3890	(133)	272	(209)	1.6*	*aaaa*	6731	(232)	828	(321)	1.4*

**WSSV (4-mer motif)**											

tata	2630	(60)	164	(128)	2.2*	tata	2630	(60)	290	(117)	2.0*
ataa^j^	3431	(74)	201	(150)	2.0*	ataa^j^	3431	(74)	327	(126)	1.7*
taaa	3538	(77)	195	(146)	1.9*	accc	1333	(88)	127	(148)	1.7*
aacc	1774	(79)	92	(142)	1.8*	taaa	3538	(77)	333	(128)	1.7*
aaaa	6450	(134)	330	(236)	1.8*	aaaa	6450	(134)	570	(210)	1.6*
accc	1333	(88)	68	(154)	1.8*	ccgg	374	(36)	33	(56)	1.6*
accg	699	(46)	33	(75)	1.6*	cccc	1348	(130)	118	(202)	1.6*
tacc	1424	(67)	64	(103)	1.5	cacg	995	(65)	86	(100)	1.5*
caac	2792	(125)	123	(190)	1.5	aacc	1774	(79)	150	(119)	1.5*
aata	4104	(89)	179	(134)	1.5	accg	699	(46)	56	(65)	1.4
tttt	6450	(160)	281	(240)	1.5	taac^j^	1888	(60)	151	(85)	1.4
taac^j^	1888	(60)	82	(90)	1.5	gtaa	1810	(58)	144	(81)	1.4
cccg	583	(56)	25	(83)	1.5	taag	1402	(45)	109	(61)	1.4
ccgg	374	(36)	16	(53)	1.5	gggt	1333	(91)	103	(124)	1.4
ccgt	940	(64)	40	(94)	1.5	tttt	6450	(160)	491	(216)	1.3

**WSSV (5-mer motif)**											

ccggg	66	(31)	7	(116)	3.8*	tataa	760	(57)	113	(151)	2.6*
tataa	760	(57)	73	(195)	3.4*	ccccc	292	(137)	41	(342)	2.5*
cccgg	66	(31)	6	(99)	3.2*	atata	716	(54)	95	(127)	2.4*
ataaa	1202	(87)	103	(263)	3.0*	ataaa	1202	(87)	147	(188)	2.2*
taaaa	1378	(99)	102	(261)	2.6*	ccggg	66	(31)	8	(66)	2.1*
aaccg	203	(44)	15	(116)	2.6*	taaaa	1378	(99)	167	(213)	2.1*
gtata	609	(67)	45	(176)	2.6*	gtata	609	(67)	73	(143)	2.1*
atata	716	(54)	51	(136)	2.5*	aaccg	203	(44)	23	(89)	2.0*
taccc	331	(75)	22	(178)	2.4*	aaaaa	2054	(142)	231	(283)	2.0*
acccg	138	(44)	9	(102)	2.3*	tatat	716	(56)	79	(110)	2.0*
aaaaa	2054	(142)	133	(325)	2.3*	acccc	320	(103)	35	(199)	1.9*
aacca	644	(96)	41	(216)	2.3*	accgg	120	(38)	13	(73)	1.9*
ccgta	208.0	(47)	13	(105)	2.2*	cccgg	66	(31)	7	(58)	1.9*
gacct	257	(58)	16	(129)	2.2*	ctcac^k^	284	(65)	30	(121)	1.9*
cgtcg	178	(59)	11	(130)	2.2*	taccc	331	(75)	34	(137)	1.8*

^a^both strands, excluding *hr*s (present for *Ac*MNPV and WSSV)

^b^expected occurrence is the occurrence of a 4-mer or 5-mer based on random distribution of nucleotides in the complete genome

^c^part of the *Ac*MNPV late initiator sequence (a/g/t)TAAG

^d^part of the *Ac*MNPV upstream activating element with sequence (a/t)GATA(a/t)

^e^part of the *Ac*MNPV downstream activating element with sequence (a/t)CACNG

^f^sequence of the *Ac*MNPV early initiator CAGT

^g^part of the CCATT box

^h^part of the Vaccinia virus late initiator sequence TAAAT and/or the intermediate initiator TAAA(a/t)

^i^sequence of the ASFV late initiator TATA

^j^part of the WSSV putative late TIS motif ATNAC

^k^part of the WSSV putative early initiator (a/c)TCANT

**Table 2:** **Consensus sequences (4–8 nt) in upstream regions of WSSV genes identified with MEME. **Only the best 3 hits of MEME are shown. In case of all WSSV genes, the number of sequences in which the consensus sequence occurred is indicated.

	**All WSSV genes (0 or 1 occurrence per individual sequence)**	**Early WSSV genes (1 occurrence per individual sequence)**	**Late WSSV genes (1 occurrence per individual sequence)**
**100 nt upstream**	gtataaaa (TATA box) (61 seqs) tgtttttt (T-rich) (65 seqs) gaggaaga (61 seqs)	gtataaaa (TATA box) ttttttca (T-rich) caacatca	gtataaaa (TATA box) tttgtgta aacc
**200 nt upstream**	gtataaaa (TATA box) (112 seqs) ttgttttt (T-rich) (65 seqs) tcctcctc (61 seqs)	agaagagg tatttttt (T-rich) agaaat	gaggaaga tcttttat (T-rich) aaaaatat (A-rich)
